# Étude transversale sur la couverture médicale des enfants porteurs de trisomie 21 au Maroc: cas de l’unité de dysmorphologie du service de pédiatrie 2 au Centre Hospitalier Universitaire Ibn Sina de Rabat

**DOI:** 10.11604/pamj.2026.53.101.49364

**Published:** 2026-02-26

**Authors:** Meriem Benadada, Saida Naji, Nada Bennani Mechita, Samia El Hilali, Yassine Sarboute, Majdouline Obtel, Asmaa Mdaghri Alaoui

**Affiliations:** 1 Équipe de Recherche sur les Anomalies Congénitales, Faculté de Médecine et de Pharmacie, Université Mohammed V, Rabat, Maroc,; 2 Laboratoire de Recherche en Compétitivité Économique et Performance Managériale, Faculté des Sciences Juridiques, Économiques et Sociales de Souissi, Université Mohammed V, Rabat, Maroc,; 3 Unité de Dysmorphologie, Service de Pédiatrie 2, Hôpital d'Enfants, Centre Hospitalier Universitaire IBN Sina, Rabat, Maroc,; 4 Laboratoire de Médecine Sociale, Laboratoire de Biostatistiques, Recherche Clinique et Epidémiologie, UPR de Santé Publique, Faculté de Médecine et de Pharmacie, Université Mohammed V, Rabat, Maroc

**Keywords:** Trisomie 21, enfants en situation de handicap, couverture médicale, coûts des soins, Down syndrome, children with disabilities, health coverage, healthcare costs

## Abstract

**Introduction:**

le syndrome de Down (trisomie 21) engendre des besoins médicaux complexes nécessitant une prise en charge pluridisciplinaire. Au Maroc, peu de données sont disponibles concernant la couverture médicale de ces patients. L'objectif de cette étude est de décrire la couverture médicale des enfants porteurs de trisomie 21 ainsi que les spécialités médicales sollicitées et les coûts générés au cours du parcours de soins de ces patients, et ce en vue d'identifier des pistes d'amélioration de leur prise en charge.

**Méthodes:**

étude transversale à visée descriptive avec un recueil de données rétrospectif, menée entre janvier 2023 et décembre 2024 auprès d'enfants suivis à l'unité de dysmorphologie de l'Hôpital d'Enfants de Rabat. Données recueillies via des fiches standardisées et des entretiens semi-directifs avec les parents.

**Résultats:**

parmi les 118 enfants inclus (52% garçons, 48% filles), la tranche d'âge prédominante était celle des 6-10 ans (31%). 58% ne disposaient d'aucune couverture médicale, exposant les familles à un financement direct. Les spécialités les plus sollicitées: endocrinologie (39,8%), gastrologie (36,4%), ophtalmologie (31,4%) et cardiologie (29,7%). D'autres, comme la psychiatrie pédiatrique (0,8%) ou la dentisterie (2,5%) restaient marginaux. Les coûts les plus élevés concernaient l'hématologie (702,5 dirhams marocains (MAD)/mois), la neurologie-développement (642 MAD/mois) et la cardiologie (413,9 MAD/mois).

**Conclusion:**

dans notre série, seuls 42% des patients disposaient de la couverture médicale. Ce constat souligne la nécessité de faire bénéficier les autres enfants atteints de trisomie 21 d'une meilleure accessibilité aux soins, d'une fluidité dans la réalisation des bilans et, plus globalement, d'un renforcement du niveau de la couverture médicale.

## Abstract (en)

**Introduction:**

Down syndrome creates complex medical needs that require multidisciplinary care. In Morocco, there is a significant scarcity of data regarding the medical coverage for these patients. This study aims to describe the medical coverage of children with trisomy 21, as well as the medical specialities involved and the costs incurred during their care pathway, with a view to identifying areas for improvement in their care, particularly in the context of the public-private partnership that is part of the process of expanding compulsory health insurance (AMO).

**Methods:**

a descriptive cross-sectional study was conducted between January 2023 and December 2024 among children followed at the dysmorphology unit of the Rabat Children's Hospital. The data were collected via standardised forms and semi-structured interviews with parents.

**Results:**

among the 118 children included (52% boys, 48% girls), the majority were aged 6-10 (31%). 58% had no medical coverage, exposing families to direct financing. The most sought-after specialities were: endocrinology/genetics (39.8%), gastroenterology (36.4%), ophthalmology (31.4%), and cardiology (29.7%). Others, such as pediatric psychiatry (0.8%) and dentistry (2.5%), remained marginal. The highest costs were for haematology (702.5 MAD/month), developmental neurology (642 MAD/month), and cardiology (413.9 MAD/month).

**Conclusion:**

in our series, only 42% of patients had medical coverage. This finding highlights the need to provide other children with Down syndrome with better access to care, smoother check-ups, and, more generally, improved medical coverage.

## Introduction

Le syndrome de Down (trisomie 21), la plus fréquente des anomalies chromosomiques autosomiques, est une affection génétique résultant de la présence d'un chromosome 21 surnuméraire. Sa prévalence mondiale est estimée entre 1 sur 600 et 1 sur 1000 naissances vivantes [1]. Bien que cette condition soit avant tout d'origine génétique, elle est loin de se limiter à une simple anomalie chromosomique: elle s'accompagne fréquemment de multiples atteintes d'organes et de troubles multisystémiques, notamment cardiaques, endocriniens, neurologiques, gastro-intestinaux, ORL, ophtalmologiques et musculosquelettiques [2]. Ces comorbidités, souvent précoces, nécessitent une prise en charge pluridisciplinaire, spécialisée et continue dès les premiers mois de vie, avec des besoins qui évoluent tout au long du développement de l'enfant. Par ailleurs, les enfants atteints de trisomie 21 (T21) présentent un profil de vulnérabilité médicale et sociale accru, justifiant des interventions coordonnées impliquant des pédiatres, généticiens, cardiologues, ORL, kinésithérapeutes, psychomotriciens, orthophonistes et autres spécialistes. Malgré une amélioration globale de l'espérance de vie des personnes atteintes de T21 grâce aux avancées médicales, les inégalités d'accès aux soins persistent, notamment dans les pays à revenu intermédiaire.

Au Maroc, malgré les efforts entrepris pour renforcer les structures de soins pédiatriques, la couverture médicale des enfants porteurs de T21 reste peu documentée et souvent insuffisante [3]. Les lacunes en matière de diagnostic précoce, de suivi multidisciplinaire et de financement des soins spécialisés exposent les familles à une charge financière et logistique significative. Compte tenu de la fréquence élevée des comorbidités associées à la T21 et de la complexité du suivi qu'elle implique, l'articulation entre les secteurs public et privé constitue une voie stratégique pour promouvoir l'équité et la durabilité du système de santé. Les conventions de coopération, la régulation des coûts des prestations médicales et la mise en place de passerelles de remboursement sont autant de leviers susceptibles de réduire la charge pesant sur les familles, tout en améliorant la qualité, la continuité et l'accessibilité aux soins de santé [3].

Dans ce contexte, l'objectif principal de cette étude est d'évaluer la couverture médicale et la charge financière, liées à la prise en charge des principales pathologies, supportées par les familles des enfants porteurs de T21, suivis à l'unité de dysmorphologie du service de pédiatrie 2 du CHU Ibn Sina de Rabat. Cette étude vise à identifier les spécialités médicales les plus sollicitées pour la prise en charge des enfants atteints de trisomie 21, les limites et insuffisances en couverture formelle et les postes de dépenses médicales les plus importants en termes de coût.

## Méthodes

**Population et type d'étude:** il s'agit d'une étude transversale à visée descriptive, avec un recueil de données rétrospectif, menée de janvier 2023 à décembre 2024 auprès des enfants suivis à l'unité de dysmorphologie de l'Hôpital d'Enfants du CHU Ibn Sina de Rabat.

### Critères d'éligibilité

**Critères d'inclusion:** ont été inclus dans cette étude les enfants porteurs de trisomie 21, âgés de 0 à 18 ans, ayant consulté en dysmorphologie entre janvier 2023 et décembre 2024 et dont les parents ont accepté de participer à cette étude.

**Critères de non-inclusion:** sur un total de 129 patients, six patients ont été exclus pour insuffisance des données recueillies et cinq pour l'injoignabilité des parents.

**Échantillonnage:** tous les enfants porteurs de T21 pris en charge dans le service durant la période d'étude, dont les parents ou tuteurs acceptent de participer à l'enquête et dont les dossiers médicaux sont disponibles et exploitables, ont été retenus. En effet, aucun calcul de la taille de l'échantillon n'a été réalisé au préalable, car notre étude avait un caractère exhaustif. Au total, 118 patients ont été colligés.

**Recueil des données:** dans le cadre de cette étude transversale, les données ont été collectées selon une approche mixte: de manière rétrospective à partir des dossiers médicaux et à l'aide de fiches d'exploitation standardisées, ainsi que directement auprès des parents et des tuteurs, au moyen d'un questionnaire structuré, administré en face à face. Les variables recueillies comprenaient les caractéristiques sociodémographiques (âge, sexe, milieu de résidence), les pathologies cliniques ainsi que les différentes spécialités sollicitées. La variable principale étudiée était le type de couverture médicale, qu'il s'agisse d'une assurance sociale ou d'un financement direct. Par ailleurs, nous avons procédé à une estimation des coûts de prise en charge des principales pathologies, analysés par spécialité médicale, en nous basant sur le prix des consultations, des explorations, des analyses et des médicaments, exprimés sous forme de dépenses mensuelles et annuelles avec un focus particulier sur le Partenariat Public-Privé (PPP).

**Analyse statistique:** une analyse descriptive a été conduite. Les informations collectées ont été saisies dans un fichier Excel afin de les analyser statistiquement à l'aide de Microsoft Office Excel. Les variables qualitatives ont été exprimées en effectifs et en pourcentage. Les variables quantitatives ont été exprimées en médiane, intervalle interquartile, car la distribution était non gaussienne.

**Considérations éthiques:** les aspects éthiques et réglementaires ont été pris en compte avant le début de cette étude. Le protocole a été soumis au comité d'éthique de la recherche biomédicale de la Faculté de Médecine et de Pharmacie de l'Université Mohamed V de Rabat, au Maroc (agrément éthique CERB 169-24/20-11-2024).

Avant l'administration des questionnaires, les parents ou les tuteurs légaux des enfants participants ont été informés des objectifs de l'étude, des procédures de collecte de données et du caractère volontaire de la participation. Un consentement éclairé a été obtenu avant l'administration du questionnaire en face à face. Pour la partie rétrospective basée sur les dossiers médicaux, l'autorisation a été obtenue auprès du chef de service. L'anonymat et la confidentialité de toutes les données collectées ont été strictement garantis.

## Résultats

**Caractéristiques démographiques et cliniques:** le Tableau 1 montre les caractéristiques sociodémographiques et cliniques des 118 patients inclus dans l'étude. L'analyse selon le sexe des enfants porteurs de T21 montrait une répartition relativement équilibrée entre les sexes, avec 61 garçons (soit 52%) et 57 filles (48%). En ce qui concerne les tranches d'âge, les enfants âgés de 6 à 10 ans constituaient la catégorie la plus représentée, avec 31% de l'échantillon (n=37). Cette tranche était suivie par les enfants de 2 à 5 ans (21%, n=25) et ceux de 11 à 15 ans (également 21%, n=25). Les enfants de moins de 2 ans représentaient 18% des cas (n=21), tandis que la tranche des 16 à 18 ans était la moins fréquente, avec seulement 9% de l'effectif (n=10).

**Tableau 1:** données démographiques, médicales et de couverture chez les patients porteurs de trisomie 21

Catégorie	Valeur n(%)
**Sexe**	-
Garçons	61 (52)
Filles	57 (48)
**Tranche d'âge**	-
<2 ans	21 (18)
2-5 ans	25 (21)
6-10 ans	37 (31)
11-15 ans	25 (21)
16-18 ans	10 (9)
**Couverture**	-
CNSS	33 (28)
CNOPS	13 (11)
FAR	4 (3)
Non couverts	68 (58)

Du point de vue de la couverture médicale, les résultats révélaient que seuls 42% (n=50) des enfants bénéficiaient d'un régime d'assurance maladie. Plus précisément, 28% (n=33) étaient affiliés à la CNSS (Caisse nationale de sécurité sociale), 11% (n=13) à la CNOPS (Caisse nationale des organismes de prévoyance sociale) et 3% (n=4) relevaient du régime des FAR (Forces armées royales). En revanche, la majorité des enfants, soit 58% (n=68), ne disposaient d'aucune couverture médicale formelle et dépendaient entièrement du financement direct assuré par leurs familles. Il est à noter qu'aucune donnée n'était manquante pour les variables sociodémographiques, cliniques et de couverture médicale. En ce qui concerne les besoins médicaux, l'analyse du Tableau 2 montre que la prise en charge des enfants trisomiques impliquait un large éventail de spécialités médicales. Les disciplines les plus sollicitées étaient l'endocrinologie et la génétique, qui concernaient 47 enfants (39,8%) dont la majorité présentait l'hypothyroïdie congénitale, suivies de près par la gastrologie avec 43 cas (36,4%), l'ophtalmologie avec 37 cas (31,4%) et la cardiologie congénitale, qui représentait 35 cas (29,7%). La neurologie et le développement venaient ensuite avec 27 enfants concernés (22,9%). Les spécialités ORL et dermatologie apparaissaient chacune dans 10 cas (8,5%), tandis que la gynécologie et l'urologie concernaient 6 enfants (5,1%). Les domaines de l'hématologie, de l'appareil locomoteur et de la posture regroupaient chacun 4 cas (3,4%). Enfin, la dentisterie (2,5%) et la psychiatrie pédiatrique (0,8%) étaient peu sollicitées.

**Tableau 2:** répartition des pathologies associées par spécialités médicales sollicitées dans la prise en charge des enfants porteurs de trisomie 21

Catégorie: spécialité	Valeur n (%)
Endocrinologie & génétique	47 (39,8)
Gastrologie	43 (36,4)
Ophtalmologie	37 (31,4)
Cardiologie congénitale	35 (29,7)
Neurologie & développement	27 (22,9)
ORL	10 (8,5)
Dermatologie	10 (8,5)
Gynécologie & urologie	6 (5,1)
Hématologie	4 (3,4)
Appareil locomoteur & posture	4 (3,4)
Dentisterie	3 (2,5)
Psychiatrie pédiatrique	1 (0,8)

**Estimation des coûts de la prise en charge par pathologie:** l'analyse des dépenses médicales repose sur les principales spécialités consultées dans le cadre de la prise en charge des enfants porteurs de T21, en lien direct avec les pathologies fréquemment rencontrées dans cette population. Tout d'abord, en hématologie, les besoins étaient dominés par la surveillance des carences martiales, nécessitant des analyses biologiques régulières. Par ailleurs, en neurologie et développement, les troubles du langage, du tonus musculaire et du développement psychomoteur imposaient un recours intensif à des prises en charge en orthophonie et en rééducation. De plus, la cardiologie occupait une place importante en raison de la prévalence élevée des malformations congénitales, telles que les canaux atrioventriculaires, qui nécessitaient des examens spécialisés (échographies, ECG) et parfois un traitement médicamenteux prolongé.

En outre, la gastroentérologie intervenait dans le suivi de troubles digestifs variés, incluant notamment la maladie cœliaque, dont le régime alimentaire associé constituait un coût notable. L'endocrinologie, quant à elle, est mobilisée principalement pour la surveillance de l'hypothyroïdie congénitale, souvent présente chez les enfants trisomiques. Enfin, l'ophtalmologie est sollicitée pour le dépistage et la correction des troubles visuels (troubles de la réfraction, strabisme), nécessitant des bilans réguliers et des prescriptions de dispositifs correcteurs. Sur la base de ces spécialités, l'analyse des coûts détaillée dans le Tableau 3 met en évidence des disparités notables selon les domaines médicaux. Les dépenses les plus importantes concernaient l'hématologie (702,5 MAD/mois; 8 430 MAD/an) en raison de la nécessité d'analyses biologiques régulières (NFS, ferritine) et du traitement associé à la prise en charge de la carence martiale. La neurologie et le développement occupaient également une place centrale (642 MAD/mois; 7 704 MAD/an), essentiellement en raison de la fréquence répétée des séances d'orthophonie (36 séances par an). En ce qui concerne la spécialité qui suit de près la neurologie et le développement, c'était la cardiologie (413,9 MAD/mois; 4 960 MAD/an) avec des charges cumulant consultations spécialisées, échographies cardiaques, ECG et traitements médicamenteux chroniques. À l'inverse, les prises en charge relativement moins coûteuses concernaient l'ophtalmologie (316,6 MAD/mois; 3 799,9 MAD/an) et l'endocrinologie (331,1 MAD/mois; 3 973,6 MAD/an), où les dépenses se limitaient essentiellement aux bilans ciblés et à des traitements substitutifs accessibles (lunettes de correction). La gastroentérologie se situait dans une position intermédiaire (441,2 MAD/mois; 5 293,8 MAD/an), marquée par les explorations de suivi et le régime sans gluten, souvent onéreux pour les familles.

**Tableau 3:** coûts de prise en charge des principales pathologies chez les enfants porteurs de trisomie 21

Domaine	Pathologie	Consultations (MAD/mois)	Explorations (MAD/mois)	Analyses (MAD/mois)	Médicaments (MAD/mois)	Total mensuel (MAD)	Total annuel (MAD)	Remarques
**Endocrinologie**	Hypothyroïdie congénitale	100	-	208,3	22,8	**331,1**	**3 973,60**	Bilan hormonal trimestriel, traitement substitutif
**Cardiologie**	Cardiopathie congénitale	133,3	141,7	50	120,9	**413,9**	**4 960,70**	Échographie, ECG, médicaments (furosémide, digoxine…)
**Hématologie**	Carence martiale	133,3	-	490	79,2	**702,5**	**8 430,40**	NFS et ferritine mensuelles, fer + vitamines
**Neurologie & Développement**	Retard de langage	600	42		–	**642**	**7 704**	Orthophonie (3 séances/mois), EEG annuel
**Ophtalmologie**	Baisse d’acuité visuelle	133,3	100,3	-	83	**316,6**	**3 799,90**	Lunettes correctrices annuelles
**Gastroentérologie**	Maladie cœliaque	133,3	166,7	121,7	19,5	**441,2**	**5 293,80**	Régime sans gluten, examens de suivi

## Discussion

Les premiers résultats mettent en évidence que dans notre série, nous avons noté une légère prédominance masculine avec 52% des cas pour 48% de sexe féminin, le sex-ratio H/F était de 1,07. Une étude réalisée à la base du programme « The Metropolitan Atlanta Congenital Defects Program » par Lary et Paulozzi [4] avait montré que les malformations congénitales étaient plus fréquentes chez les nourrissons de sexe masculin avec une prévalence de 3,9% contre 2,8% chez les sujets de sexe féminin. Les résultats obtenus étaient concordants avec ce qui a été décrit dans la littérature. La tranche d'âge la plus représentée dans notre étude est celle de 6 à 10 ans. Toutefois, il reste difficile d'établir des comparaisons strictes, car l'évolution des besoins médicaux chez les enfants porteurs de T21 variait avec l'âge. En effet, si les consultations sont très rapprochées durant les premières années de vie en raison des multiples bilans nécessaires, elles tendent à devenir annuelles après l'âge de trois ans. Néanmoins, à partir de 6 ans, une augmentation de la fréquence peut s'observer, car de nouveaux problèmes médicaux et scolaires commencent souvent à apparaître, ce qui justifie une surveillance renforcée dans cette tranche d'âge, comme le souligne également la littérature [5].

La couverture médicale demeure largement insuffisante pour les enfants atteints de T21. En effet, 58% d'entre eux ne bénéficiaient d'aucune assurance maladie formelle, contraignant ainsi leurs familles à prendre en charge directement les frais de santé, ce qui constituait un facteur de vulnérabilité économique important. Ce taux est significativement inférieur à celui observé au niveau national: selon les Indicateurs sociaux du Maroc 2023 publiés par le Haut-Commissariat au Plan, le taux de couverture médicale de la population marocaine atteint 70% [6]. Cette situation peut s'expliquer en partie par la fin du régime d'assistance médicale (RAMED) [7], survenue en décembre 2022. Le RAMED, instauré par la loi 65-00 [8], était un dispositif de protection sociale destiné aux populations économiquement vulnérables, visant à faciliter leur accès aux soins de santé. Sa suppression s'inscrit dans le cadre d'une réforme globale de la couverture sociale au Maroc, ayant conduit à son remplacement progressif par l'assurance maladie obligatoire (AMO), désormais gérée par la CNSS [9]. Toutefois, la transition vers l'AMO n'a pas permis de couvrir une grande partie des populations vulnérables, désormais exclues de la gratuité et contraintes à une couverture contributive souvent inabordable.

Au-delà des implications financières, l'absence de couverture médicale peut également avoir des conséquences directes sur la santé et la survie des enfants concernés. Une étude menée aux États-Unis sur des nouveau-nés présentant une malformation cardiaque congénitale a révélé que les enfants non couverts par une assurance médicale présentaient un risque de décès trois fois plus élevé que celui de ceux bénéficiant d'une assurance privée [10]. Ce constat met en lumière le rôle majeur et déterminant de la couverture médicale dans l'amélioration des chances de survie de cette population particulièrement vulnérable, notamment les enfants porteurs de T21, chez qui les malformations cardiaques sont fréquentes. Par ailleurs, l'analyse détaillée des besoins médicaux mobilisés souligne l'ampleur du recours à une prise en charge multidisciplinaire, ce qui implique un large éventail de spécialités médicales, soulignant la complexité de leur état de santé. En effet, la majorité des enfants de notre cohorte nécessitaient un suivi en endocrinologie et en génétique, soit 39,8% des cas. Cette forte proportion s'expliquait par la fréquence des troubles hormonaux et des altérations du développement staturo-pondéral classiquement associés à la T21. Ces résultats rejoignaient ceux rapportés dans la littérature. Déjà en 1975, Davis avait mis en évidence une prévalence accrue d'anomalies du fonctionnement thyroïdien chez les enfants porteurs de T21 [11]. Par la suite, plusieurs auteurs ont confirmé ces observations, rapportant une fréquence avoisinante de 37% de cas présentant des troubles thyroïdiens [12]. Ensuite, la gastrologie intervenait dans 36,4% des situations, souvent en lien avec des anomalies digestives, tandis que la cardiologie congénitale concernait 29,7% des enfants en raison de la forte occurrence de malformations cardiaques dans cette population. D'ailleurs, selon les rapports internationaux, les maladies coronariennes touchent 40% à 63% des personnes atteintes du syndrome de Down et constituent un facteur clé de morbidité et de décès prématuré chez ces personnes [13-16].

L'ophtalmologie (31,4%) et la neurologie associée au développement psychomoteur (22,9%) apparaissaient également comme des spécialités fortement sollicitées, traduisant la nécessité d'un suivi sensoriel et neurodéveloppemental structuré et durable [17]. L'impact économique de cette prise en charge multidisciplinaire était encore plus frappant lorsqu'on considérait les enfants cumulant plusieurs pathologies chroniques. À titre d'exemple, un enfant atteint simultanément d'une cardiopathie congénitale, d'une hypothyroïdie congénitale et nécessitant une correction ophtalmologique représentait une charge directe estimée à près de 12 734 MAD par an (soit environ 1 062 MAD par mois). Ce montant, qui ne prenait pas en compte les frais indirects (transport, pertes de revenu parental, complications éventuelles), illustre la vulnérabilité économique des familles, particulièrement lorsqu'elles ne disposent d'aucune couverture médicale. Une telle situation renforce la nécessité d'un soutien institutionnel accru et d'une organisation adaptée des soins, avec un focus sur le PPP comme levier pour réduire le poids des dépenses supportées par les ménages.

Toutefois, certaines disciplines, pourtant essentielles dans l'accompagnement global de l'enfant, restaient très peu mobilisées. C'est notamment le cas de la psychiatrie pédiatrique, sollicitée chez seulement 0,8% des enfants, et de la dentisterie, observée dans 2,5% des cas. Cette faible représentativité pourrait être le reflet d'un double phénomène : d'une part, une sous-évaluation des besoins liés à ces disciplines et, d'autre part, un accès limité à ces services spécialisés. Or, la santé mentale et l'hygiène bucco-dentaire jouaient un rôle central dans la qualité de vie et le développement global des enfants atteints de T21 [18,19].

S'agissant d'une enquête transversale, nos résultats concordaient généralement avec ceux d'autres études et reflétaient essentiellement la situation observée dans cette cohorte à un moment donné. Ils ne doivent donc pas être interprétés comme la prévalence réelle de ces pathologies chez l'ensemble des enfants porteurs de T21. L'échantillon, bien que ciblé et exhaustif pour la période étudiée, reste modeste et monocentrique, ce qui limite la généralisation des résultats à l'échelle nationale et n'autorise pas de comparaison avec un groupe de contrôle. De plus, certains biais méthodologiques doivent être pris en compte : un biais de sélection, lié à l'inclusion exclusive des enfants suivis dans un centre de référence; un biais d'information, dû au caractère parfois incomplet des dossiers médicaux; et un biais de mémorisation, lors des entretiens avec les parents concernant les dépenses médicales. Malgré ces limites, l'étude apporte un éclairage précieux sur les disparités d'accès aux soins et les défis financiers auxquels font face les enfants trisomiques au Maroc et plaide pour une réforme renforcée de la couverture médicale ainsi qu'une meilleure organisation de la prise en charge multidisciplinaire, incluant un PPP en soutien aux familles.

## Conclusion

Cette étude, menée auprès de 118 enfants porteurs de T21 suivis au CHU Ibn Sina de Rabat, a mis en évidence une couverture médicale largement insuffisante, exposant la majorité des familles à un financement direct des soins. En conséquence, celles-ci supportent une charge économique importante, particulièrement dans le cadre de la prise en charge multidisciplinaire requise par les comorbidités fréquentes. De plus, certaines disciplines essentielles, telles que la santé mentale et l'hygiène bucco-dentaire, sont restées très peu sollicitées, ce qui souligne la nécessité de renforcer leur intégration dans le parcours de soins. Ainsi, nos résultats montrent que, dans ce contexte spécifique, l'absence de couverture adaptée aggrave la vulnérabilité des familles et limite l'accès à des soins spécialisés réguliers. Dans cette perspective, la mise en place d'un « parcours T21 » représenterait une avancée majeure en garantissant une prise en charge coordonnée et continue, depuis le diagnostic jusqu'à l'âge adulte, couvrant à vie les soins de base et spécialisés et allégeant la charge financière et psychologique des familles. Par ailleurs, le renforcement du réseau de centres spécialisés demeure essentiel pour améliorer l'accessibilité et la coordination des soins.

### Etat des connaissances sur le sujet

- La T21 est l'anomalie chromosomique autosomique la plus fréquente, avec une prévalence mondiale estimée entre 1/600 et 1/1000 naissances vivantes et elle s'accompagne de multiples comorbidités nécessitant une prise en charge pluridisciplinaire précoce et continue;
- Les enfants porteurs de T21 présentent une vulnérabilité médicale et sociale accrue, justifiant des interventions coordonnées impliquant plusieurs spécialités médicales et paramédicales;
- Au Maroc, la couverture médicale des enfants atteints de T21 reste insuffisamment documentée, malgré les efforts entrepris pour renforcer les structures de soins pédiatriques.

### Contribution de notre étude à la connaissance

- Cette étude montre que, parmi 118 enfants porteurs de T21 suivis sur deux ans à l'Hôpital d'Enfants du CHU Ibn Sina de Rabat, 58% ne bénéficiaient d'aucune couverture médicale, exposant ainsi leurs familles à un financement direct important;
- Elle met en évidence que les spécialités les plus sollicitées étaient l'endocrinologie/ génétique (39,8%), la gastro-entérologie (36,4%), l'ophtalmologie (31,4%) et la cardiologie (29,7%);
- Elle révèle que les postes de dépenses les plus élevés concernaient l'hématologie (702,5 MAD/mois), la neurologie-développement (642 MAD/mois) et la cardiologie (413,9 MAD/mois), soulignant le poids économique considérable de la prise en charge multidisciplinaire.
